# Potential lethal outbreak of coronavirus disease (COVID-19) among the elderly in retirement homes and long-term facilities, France, March 2020

**DOI:** 10.2807/1560-7917.ES.2020.25.15.2000448

**Published:** 2020-04-16

**Authors:** Jean-François Etard, Philippe Vanhems, Laëtitia Atlani-Duault, René Ecochard

**Affiliations:** 1 TransVIHMI, Institut de Recherche pour le Développement (IRD), Institut National de la Santé et de la Recherche Médicale (INSERM), Université de Montpellier, Montpellier, France; 2 EpiGreen, Paris, France; 3 Hospices Civils de Lyon, Service d‘Hygiène Hospitalière, Epidémiologie, Infectiovigilance et Prévention, Lyon, France; 4 Laboratoire des Pathogènes Emergents, Centre International de Recherche en Infectiologie (CIRI), Université de Lyon, Lyon, France; 5 Centre Population et Développement (CEPED), Institut de Recherche pour le Développement (IRD), Institut National de la Santé et de la Recherche Médicale (INSERM), Université de Paris, Paris, France; 6 Fondation Maison des Sciences de l’Homme (FMSH), Paris, France; 7 Laboratoire de Biométrie et Biologie Évolutive, Équipe Biostatistique Santé, Pierre-Bénite, France; 8 Hospices Civils de Lyon, Service de Biostatistique-Bioinformatique, Lyon, France; 9 Université de Lyon, Lyon, France

**Keywords:** COVID-19, elderly, long-term care facilities

## Abstract

Motivated by the potential devastating effect of a COVID-19 outbreak in retirement homes and long-term facilities for dependent elderly, we present the impact of worst-case scenarios in French institutions using a specific age structure and case–age fatality ratios. The death toll could equal the yearly death toll caused by seasonal influenza in those older than 65 years or could largely exceed that, depending on the final attack rate and proportion of infected institutions.

Reports from the coronavirus disease (COVID-19) outbreak in China identified older age as a predictor of severity and mortality [1]. A retrospective analysis of individual case data from China and elsewhere showed a very strong age gradient in the infection fatality ratio (IFR) and case fatality ratio (CFR) [2]. In France, as at 15 March 2020, people older than 75 years accounted for 20% of the confirmed cases but 79% of the deaths [3]. Cardiovascular diseases, hypertension and diabetes mellitus were the comorbidities most frequently associated with COVID-19, and many patients died of their original comorbidities [4,5]. All these comorbidities are highly common among dependent elderly people housed in institutions. Using simple calculation, we present here the impact of several worst-case scenarios in French institutions.

## Potential number of deaths

We studied different scenarios to simulate the potential death toll of an outbreak of COVID-19 in the French institutions in the absence of mitigation measures. We extracted the age structure of residents from 40 “Habitat et Humanisme” institutions for dependent elderly people. In France, at the end of 2017, 766,400 people above 60 years of age were housed in retirement homes for dependent elderly people, in long-term care facilities or received home-based care [6]. While the calculation of IFR is based on the proportion of all infected cases, i.e. a precise estimation of the proportion of symptomatic and asymptomatic cases, we restricted our analysis to CFR. The CFR, estimated from China and elsewhere, for 60–69 year-olds, 70–79 year-olds and those 80 years and older were, respectively, 3.6%, 8.0% and 14.8% [2]. We applied the hypothesis that 30–50% of the institutions were infected, a final attack rate (AR) of 30–50% among the residents and applying age-specific fatality ratios, with their confidence intervals (CI), from [2]. By the time the epidemic is over, the resulting death toll in these institutions could be equal to the yearly national death toll caused by seasonal influenza in those older than above 65 years (9,025 deaths on average between 2000 and 2009) or it could largely exceed that figure, depending on the final AR and the proportion of infected institutions (Table) [7]. Among this frail population, part of this excess in mortality may be due to the acceleration of underlying unavoidable process and another part directly attributable to the infection.

**Table:** Potential number of coronavirus disease (of COVID-19) deaths in institutions for dependent elderly people, by proportion of infected institutions and final attack rate among residents, France, March 2020 (n = 766,400 residents)

Attack rate among residents	Percentage of infected institutions					
	30%		40%		50%	
	n	95% CI	n	95% CI	n	95% CI
30%	8,682	7,283–10,283	11,576	9,710–13,711	14,470	12,138–17,138
40%	11,576	9,710–13,711	15,435	12,947–18,281	19,294	16,184–22,851
50%	14,470	12,138–17,138	19,294	16,184–22,851	24,117	20,230–28,564

CI: confidence interval.

Age distribution of residents, based on 40 “Habitat et Humanisme” institutions: 60–69 years: 2.85%; 70–79 years: 11.5%; ≥ 80 years: 85.7% (personal communication: Emile Hobeika, March 2020).

We used the age-stratified case fatality ratios and their 95% CI from [2].

## Discussion

In a given institution, the mortality caused among residents by an outbreak of acute respiratory infections (ARI) or lower respiratory tract infections depends on the final AR and the CFR. An AR of 25% among residents is common and ARI are already the main cause of death from infectious aetiology in institutions [8-10]. In France, three-quarters of residents suffered from a least one cardiovascular disease (mainly hypertension), 42% from a dementia and 18% from a broncho-pulmonary condition [11].

The population estimate of the basic reproductive number R_0_ of severe acute respiratory syndrome coronavirus 2 (SARS-CoV-2) during the early stage of the outbreak in China was 2.2 (90% high-density interval: 1.4–3.8) with a much higher probability of superspreading events than in seasonal influenza [12]. Given the lack of previous immunity, the circulation of the virus in the general population, the intensity of contacts between staff members in institutions, the underlying comorbidities of the residents and the lack of antiviral treatment, the R_0_ could be much higher in institutions than in the general population.

As in other health emergencies, elderly people are often the invisible part of the crisis [13]. Dependent elderly people are more prone to atypical clinical presentations [14]. Atypical respiratory viral infections presentations, combined with the challenge to properly conduct an interview in case of neurocognitive disorders, will delay diagnosis and treatment. This will be detrimental to the individual prognosis and will facilitate viral spread within the institution. Since confirmation tests are not systematically done, the precise final death toll attributable to COVID-19 in institutions will remain largely unknown.

Of course, the risk of a given institution to be affected will depend on its location and the timing of the outbreak. A range of measures have been recommended in France to reduce the risk of introduction of SARS-CoV-2 in institutions caring for elderly people and to reduce the risk of nosocomial transmission: lockdowns, suspension of visits and personal aids, secured supply chains, isolation of cases, extended barrier measures, sanitation, limitation of internal activities, etc [15]. Social isolation will in turn increase the risk of cognitive disorders and further delay diagnosis [16]. Close contacts between residents and nursing staff and frequent contacts within nursing staff, lead to a high probability of infection among the nursing staff. As a consequence, the institution needs to hire temporary staff and organise rotations during a long period of heavy workload. The institutions then need to cope with a double burden: a high burden of disease among residents and severe staff constraints. 

Public information and communication campaigns should be channelled to protect the most vulnerable and oldest people of our society, to make them visible and to provide strong psychological support to the nursing staff. In addition, we also need to strengthen the communication between nursing staff and families at the end of a resident’s life as well as after death. In order to release the pressure on general hospitals, a palliative approach of care should be offered within the impacted institutions, taking into account ethical considerations.
